# Time until exit from paid work after ages 65–69 and ≥ 70, respectively: importance of prior sickness absence and disability pension

**DOI:** 10.1186/s12889-025-24425-1

**Published:** 2025-09-09

**Authors:** Aleksiina Martikainen, Kristina Alexanderson, Pia Svedberg, Kristin Farrants

**Affiliations:** Division of Insurance Medicine, Department of Clinical Neuroscience, Karolinska Institutet, 171 77 Stockholm, Sweden

**Keywords:** Extended working life, Mental, Sick leave, Somatic, Retirement

## Abstract

**Background:**

As populations age, more knowledge is needed on people who extend their working lives. The aim of this study was to explore if prior sickness absence (> 14 days) and/or disability pension (SADP) in mental and/or somatic diagnoses were associated with time until work exit after ages 65–69 and ≥ 70, respectively, among women and men.

**Methods:**

This prospective population-based cohort study included all 65–69-year-olds (cohort65, n = 201,263) and ≥ 70-year-olds (cohort70, n = 93,751) who were in paid work in Sweden in 2014. SADP was measured in 2010–2014 as yes/no and categorised by number of days. Work was defined as work income ≥ 75% of the income requirement for SA benefits. Work exits were observed between 2015 and 2018. Linked microdata were analysed using Accelerated Failure Time model to derive Time Ratios (TR) and 95% confidence intervals (95%CI), adjusting for sociodemographic factors and branch of industry, and censoring for death and emigration.

**Results:**

Most individuals had no prior SADP (cohort65: 66.3% women, 75.8% men; cohort70: 96.8% women, 97% men). In both cohorts and sexes, ~ 80% remained in paid work at least some time during follow-up, and ~ 41% worked throughout the follow-up. In cohort65, women with prior mental SADP (TR 0.94; 95%CI 0.93–0.96), and women (0.95; 0.94–0.96) and men (0.94; 0.93–0.95) with prior somatic SADP had marginally shorter time until work exit than individuals of the same sex without the corresponding SADP. Prior SA was generally not associated with work exit in cohort70.

**Conclusions:**

Neither mental nor somatic SADP was strongly associated with time until work exit.

**Supplementary Information:**

The online version contains supplementary material available at 10.1186/s12889-025-24425-1.

## Background

Labour market participation among older individuals has increased over the past decade in many OECD countries and is expected to continue to increase in the future [1–3]. This upward trend is driven by various factors such as ageing populations, increased life expectancy [3] and healthy life years [2], as well as policy reforms aimed at promoting extended working lives [4–6]. Extended working lives are increasingly recognized as a necessary response to the challenges of rapidly ageing populations [2]. Consequently, incentives are needed to encourage people to work longer, support employers to retain and hire older workers, and promote fulfilling careers across the lifespan [2].

In Sweden, there is no fixed retirement age, but there has been a relatively strong social norm to retire at age 65 [7]. However, this norm is gradually becoming weaker [8]. There has been a rapid increase of people in paid work after age 65, growing from < 10% in 1995 to 24% in 2010 [9]. Many of those who work after age 65 in Sweden work part-time, make temporary incursions into work [10] or are self-employed [3, 11]. Also, many people take out old-age pension while simultaneously having income from work [10]. The Swedish system also allows a person to simultaneously be in paid work, receive partial sickness absence (SA) and, if aged below 65, partial disability pension (DP). Thus, rather than being a clear-cut, one-time event, retirement is today understood as a gradual process towards exit from work. This process can take several years and contain many phases such as bridge employments, unsalaried periods of non-employment, and re-retirement [3].

While a considerable body of research has focused on people who leave the labour market before reaching the standard retirement age, less is known about people who continue working beyond it. Furthermore, there are almost no studies on individuals who work after age 70. Prior studies have identified certain demographic and socio-economic factors associated with those who prolong their working lives. For example, several studies have shown that men are more likely to extend their working lives than women [12–15]. Furthermore, the likelihood of working after the standard retirement age has been found to be higher among individuals who have higher education [11, 12, 14, 16], are born in Sweden [12], have children living at home [12], and who live in big cities [12, 14].

Besides sociodemographic factors, continuing in paid work is also affected by work environment and work demands [17], which vary greatly across occupations and industries. Prior research indicates that individuals in physically demanding jobs tend to retire earlier [18], whereas those in higher occupational classes [19], non-manual jobs [20] and in occupations with high work control [21] are more likely to retire later than the standard retirement age. Additionally, those who report lower work pace, recognition from management, and opportunities for influence and development have been found to be more likely to extend their working lives [18]. Some studies also suggest that reduction to part-time work at older age can have positive health effects [22]. However, access to work adaptions like flexible or reduced working hours varies greatly, with the poorest access being observed among women and those with shorter education and reduced work ability [23]. Therefore, it is important to consider occupational and industrial differences as type of work can substantially influence the feasibility of extended working life.

While sociodemographic factors and occupational conditions play an important role for extended working life, health also emerges as a central factor. The healthy worker effect suggests that people who work are often healthier than people who do not work, which seems to hold true for people aged below 64 [24–26]. However, it is unclear whether this applies to people aged above 64, although older people are more likely to have different health issues [27, 28]. A recent systematic review [29] found that most of the included studies (42 of 66) showed better health among people who worked after age 64 compared to non-workers, while 21 studies reported insignificant associations, and six studies found better health among non-workers. Overall, no conclusive scientific evidence could be established regarding the role of health for paid work beyond age 64, largely due to the limited quality and considerable heterogeneity among the studies (e.g., differing definitions of work, different study populations, and different measures of health). The association between health and retirement is complex and possibly influenced by factors such as work capacity [30], financial situation, and labour market conditions.

Sometimes, morbidities lead to temporarily or permanently reduced work capacity, which can be operationalised by information about sickness absence (SA) and disability pension (DP). In the traditional working age population (up to age 64), prolonged SA and DP can substantially impact an individual’s career opportunities, personal economy, and lifestyle [31], and also have a broader influence on organizations and societies. Several studies have shown that poor health increases the risk of leaving labour market before reaching standard retirement age, mainly through DP [32–36]. Meanwhile, it is unclear if and how prior SA and DP are associated with labour market exit after the standard retirement age. Currently, only a few, primarily explorative studies are available, all from Sweden. These studies have shown, for example, that individuals who continued working after age 65 had fewer SA days before 65 than non-workers, and that their own SA days decreased after 65 [37]. Further, SA rates declined among people aged over 65 between 1995 and 2010 [9]. SA in mental diagnoses is far less common after age 65 than among younger people [39]. History of SA and DP have both been linked to lower likelihood of being in paid work after age 65 [12, 14]. For SA, history of mental diagnoses seem to decrease the odds more than somatic diagnoses, while people with DP seem to have low odds regardless of the diagnoses [12]. Given the differences between mental and somatic SA, such as longer length of mental SA compared to other SA spells on average [38, 39] and stigma related to mental diagnoses [40–42], it is important to examine these diagnoses separately. Further, it is important to investigate the associations of SA and DP separately for women and men, as women have been found to have higher rates of SA and DP than men [43], particularly in Sweden [44], both within the working-age population [44] and among workers aged over 60 [12, 14]. Thus, the aim of this study was to examine if prior sickness absence and/or disability pension (SADP) in mental and/or somatic diagnoses were associated with time until work exit after ages 65–69 and ≥ 70, respectively, among women and men.

## Methods

This study was a four-year prospective population-based cohort study of 201,263 individuals aged 65–69 in 2014, and 93,751 individuals aged ≥ 70 in 2014. We examined the association between prior sickness absence and/or disability pension (SADP) in mental and/or somatic diagnoses (2010–2014) and time until work exit after ages 65–69 and ≥ 70, respectively (2015–2018), among women and men. In this study, ‘time until work exit’ refers to the time until leaving *paid* work, including any type of work that generates taxable income. Although this study specifically focused on paid work, it is important to acknowledge that ‘work’ is a broad concept, also including unpaid work such as unpaid caregiving, volunteering, or internships [45].

### Social Insurance system in Sweden

The Swedish public sickness absence insurance system covers all individuals with work income, parental-leave allowance, and unemployment benefits. All people who fulfil the minimum annual income requirement of 24% of the price base amount (a government-set amount that is used to calculate various public benefits and annually adjusts for inflation) can receive SA benefits in case of reduced work capacity due to disease or injury [46, 47]. After the first seven self-reported SA days, a physician certificate, including information on diagnosis, is required for further SA. A salary deduction is made on the first waiting day, whereafter SA benefits cover up to 80% of lost income up to a certain level. Employers provide sick pay during days 2–14 for employees, after which the Social Insurance Agency provides SA benefit. Since we utilised data from Social Insurance Agency in our study, we only use information about SA spells that exceeded 14 days. Disability pension (DP), in turn, can be granted to people aged 19–64 in case of permanently reduced work capacity due to disease or injury. DP covers about 65% of lost income up to a certain level. Both SA and DP can be granted for part- or full-time: 25, 50, 75, or 100% of ordinary working hours.

During the time of this study, there was no maximum limit for the length of SA for people aged below 65. When aged 65–69, there was a maximum limit of 180 SA days in total, but it was possible to be granted additional SA days if the Social Insurance Agency found it plausible that the person would return to work. From the age of 70, there was a limit of 180 SA benefit days in total, whereafter no more SA benefit could be granted.

### Old-age pension system in Sweden

Sweden has no fixed retirement age, but rather a flexible pension system that allows individuals to choose when to start drawing their pension. The social norm to retire at age 65 [7] has recently started to weaken [8], resulting in a wide age span for pension withdrawals. During the period covered by this study, individuals could begin to take out public old-age pension from age 61, either partially or fully, or delay it indefinitely. There are no restrictions on combining any level of paid work with a full- or partial old-age pension. Thus, people can have income from both work and pension simultaneously [48]. The Swedish old-age pension system thus facilitates continued paid work beyond the normative retirement age, particularly for those working part-time. During the study period, most employees had the legal right to keep their permanent positions until age 67. After age 67, job security declined as the Employment Protection Law no longer applied, leaving it up to employer to decide whether to retain workers older than 67. Nonetheless, many individuals continue working beyond this age, encouraged by societal initiatives such as tax reductions on work income for those over 67.

### Study population

The source population included all individuals who lived in Sweden and were aged 65–69 (cohort65) and ≥ 70 (cohort70), respectively, at baseline in 2014. We wanted to study individuals who were actively engaged in any level of paid work at baseline in 2014. Thus, we only included individuals who:

- lived in Sweden throughout 2010–2015,
- were in paid work at baseline in 2014,
- did not have more than 75% DP in 2014,
- were not on full-time DP in 2013,
- had not been on full-time SA for two years as of 31 December 2014, and
- did not have SA and/or DP that alone or together exceeded 75% in both 2013 and 2014.

**Fig. 1:**
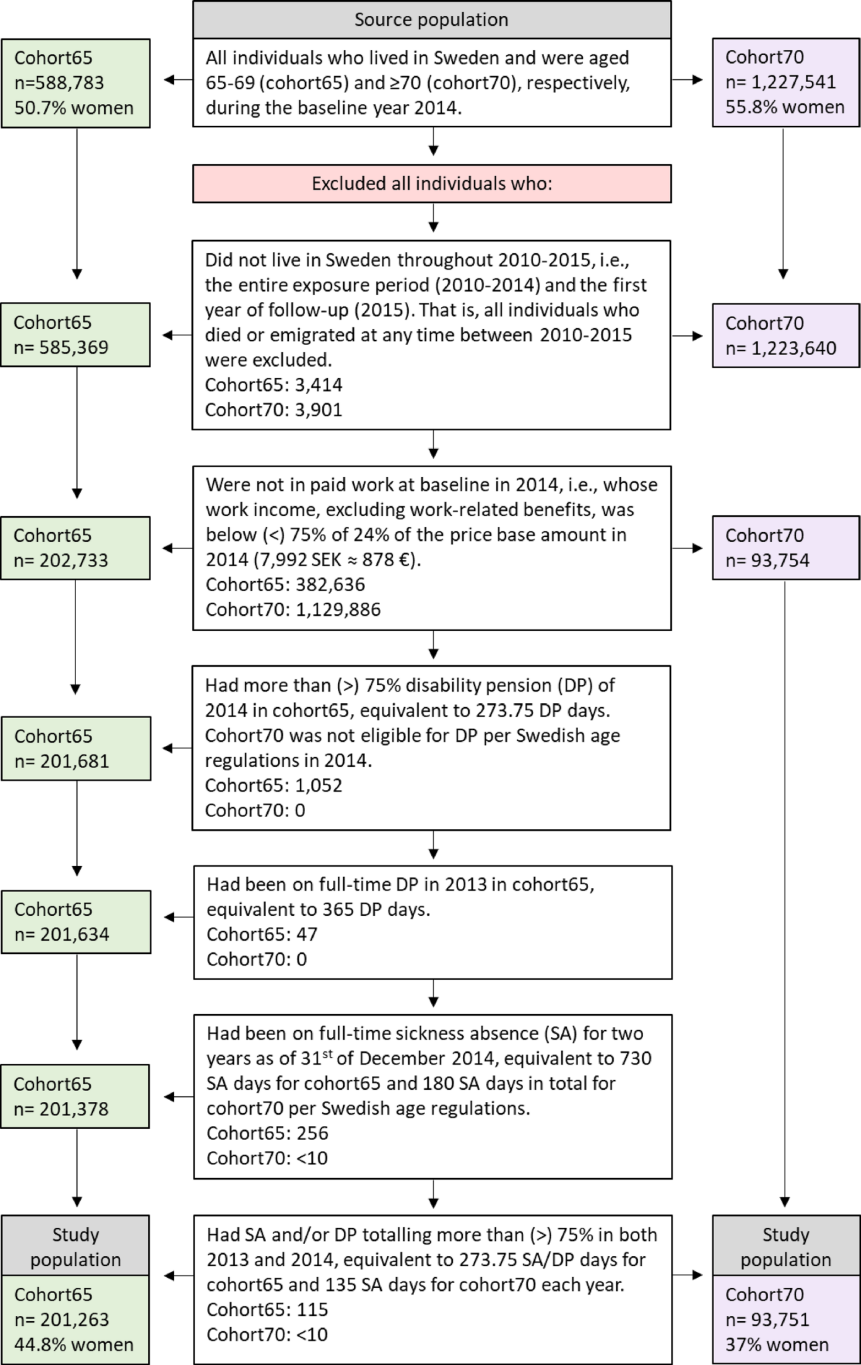
Flowchart illustrating the inclusion criteria

After applying the inclusion criteria, the study population consisted of 201,263 individuals aged 65–69 (cohort65, 44.8% women) and 93,751 individuals aged 70 or older (cohort70, 37% women). Additional details about the inclusion criteria are presented in Fig. 1.

### Data sources

This study utilized microdata derived from the following three nationwide registers in Sweden, linked at the individual level:

- Data on sociodemographic and work-related factors, as well as information on income and emigration were obtained from the Statistics Sweden’s Longitudinal Integrated Database for Health Insurance and Labour Market Studies (LISA) [49].
- Information on SA and DP, including the main diagnosis according to the International Classification of Diseases (ICD-10) [50], was derived from the Social Insurance Agency’s Micro Data for the Analysis of Social Insurance (MiDAS) [51].
- Death years were retrieved from the National Board of Health and Welfare’s Cause of Death register.

### Exposures

The exposure variable was SA (spells > 14 days) and/or DP (hereafter SADP), in 2010–2014. SADP was grouped based on the main diagnosis:

- mental diagnoses (ICD-10: F00-F99 and Z73), referred to as mental SADP
- somatic diagnoses (ICD-10: all other diagnoses, including missing information on diagnoses), referred to as somatic SADP. In total, the information on diagnoses was missing for 704 individuals with SA and 955 individuals with DP
- all diagnoses, i.e., both mental and somatic diagnoses, referred to as all-cause SADP

Within these diagnosis-groups, two types of SADP measures were utilized:

- a binary variable (yes/no, regardless of the number of days), and
- total number of SADP net days during the five-year exposure interval (2010–2014). Net days were used to make SA and DP gross days of different degrees (25%, 50%, 75%, and 100%) comparable and correspond to full-time. For instance, 10 gross days of 50% absence were converted to 5 net days of 100% absence. The number of net days were categorized as follows:
  - 0, > 0–30, > 30–90, > 90–180, > 180–365, > 365–731, or > 731 net days for cohort65 (aged 65–69 in 2014).
  - 0, > 0–30, > 30–90, > 90–180, or > 180 net days for cohort70 (aged ≥ 70 in 2014).

This categorization was based on the observed distribution of SADP days within each cohort, which was influenced by Swedish age regulations for SA and DP. For instance, individuals in cohort70 were not eligible for DP during the exposure interval, as DP was not possible when aged 65 or above.

### Outcome

The outcome was time until work exit, measured in years. Work exits were observed between 2015 and 2018, and considered to occur at the first year when an individual’s annual work-related income fell below 75% of the income requirement for SA benefits, which, in turn, was 24% of the price base income, explained before. Thus, an individual was considered to have exited from paid work when their annual work-related income fell below 7974–8190 SEK ≈799–856 EUR depending on the outcome year. Work-related income included income from employment, self-employment, and work-related benefits from the National Social Insurance Agency (such as SA benefits and parental-leave allowance). This income limit was rather low in order to capture the full spectrum of older workers with different levels of income; even those who worked part-time, had low income, or who had been on long-term SA or DP (which in most cases cover 75% of lost income).

### Covariates

Covariates from the baseline year 2014 included sex (woman or man), age (65, 66, 67, 68, or 69 for cohort65; 70–74 or ≥ 75 for cohort70), educational level (elementary (0–9 years), high school (9–12 years), or university/college (> 12 years)), birth country (Sweden or other than Sweden), living area (large city (Stockholm, Gothenburg, Malmö), medium-sized city (> 90,000 inhabitants within 30 km of municipal centre), or small city/village (< 90,000 inhabitants within 30 km of municipal centre))[52], partnership status (married/cohabiting or single), and branch of industry (manufacturing, services, hospitality, transports, construction and installation, care and education, or missing information).

### Statistical analysis

Descriptive analyses were conducted in which SA and DP were examined both separately and combined (SADP). The combined measure of SADP was used as an exposure variable the main analyses, which were conducted with Accelerated Failure Time (AFT) model with Weibull distribution [53]. Crude and adjusted time ratios (TR) with 95% confidence intervals (95% CI) were calculated for the association between prior SADP (2010–2014) and time until work exit (2015–2018). SADP was analysed separately in the following diagnosis-groups:

1. mental SADP (reference group: no mental SADP, i.e., no SADP or only somatic SADP),
2. somatic SADP (reference group: no somatic SADP, i.e., no SADP or only mental SADP), and
3. all-cause SADP (reference group: no SADP)

Within these three diagnosis-groups, SADP was analysed as a binary variable (yes/no) in one model and categorised based on the total number of SADP net days during the exposure interval in another model. Analyses were conducted separately for cohort65 and cohort70, stratifying by sex within the respective cohort. The analyses were adjusted for covariates in a stepwise matter. First, crude estimates were calculated for the exposure variable and each covariate. Secondly, analyses were adjusted for age, sex (except in the sex-stratified analyses), educational level, birth country, living area, and partnership status (Model 1). Finally, the analyses were additionally adjusted for branch of industry (Model 2).

Further, sensitivity analyses were conducted since missing diagnoses were coded as somatic diagnoses in the main analysis, but some of the missing diagnoses could have been mental diagnoses. In the sensitivity analyses, individuals with SA in missing diagnoses (n = 640 in cohort65, n = 64 in cohort70), and individuals with DP in missing diagnoses (n = 955 in cohort65, n = 0 in cohort70 due to Swedish age regulations for DP) were excluded instead of coding these cases as somatic diagnoses. Results from the sensitivity analyses were almost identical to the results obtained from the main analyses. All statistical analyses were conducted with RStudio version 4.3.1.

## Results

Characteristics of the study cohorts are presented in Table 1. There were more men in both cohorts, especially in cohort70. The majority were born in Sweden in both cohorts. The proportion of individuals who had been on prior SADP was clearly higher in cohort65 (~ 34% of women, ~ 24% of men) compared to the cohort70 (~ 3% of women and ~ 3% of men). In both cohorts, it was more common to have been on somatic SADP (cohort65: ~ 27% of women, ~ 22% of men; cohort70: ~ 3% of women and ~ 3% of men) than mental SADP (cohort65: 4% of women, ~ 2% of men; cohort70: 0.1% of women and 0.1% of men). Approximately 80% remained in paid work at least some time during the follow-up across cohorts and sexes. Participation in paid work decreased gradually in both cohorts during the follow-up, while ~ 41% worked throughout the follow-up across cohorts and sexes.

**Table 1:** Descriptives of sociodemographic and work-related factors, sickness absence (SA), disability pension (DP), and paid work

Variables in 2014	Cohort65 (aged 65–69 in 2014)						Cohort70 (aged ≥ 70 in 2014)					
	All (n=201,263)		Women (n=90,100)		Men (n=111,163)		All (n=93,751)		Women (n=34,723)		Men (n=59,028)	
	n	%	n	%	n	%	n	%	n	%	n	%
Sex												
Women	90,100	44.8					34,723	37.0				
Men	111,163	55.2					59,028	63.0				
Mean age in 2014 (SD)	66.6 (1.39)		66.5 (1.37)		66.6 (1.40)		73.0 (3.43)		72.8 (3.24)		73.2 (3.52)	
Educational level												
Elementary^a^	41,966	20.9	14,011	15.6	27,955	25.1	25,447	27.1	7,736	22.3	17,711	30.0
High school	82,248	40.9	37,506	41.6	44,742	40.2	35,276	37.6	13,406	38.6	21,870	37.1
University/College	77,049	38.3	38,583	42.8	38,466	34.6	33,028	35.2	13,581	39.1	19,447	32.9
Birth country												
Sweden	181,876	90.4	80,623	89.5	101,253	91.1	86,861	92.7	32,056	92.3	54,805	92.8
Outside Sweden^b^	19,387	9.6	9,477	10.5	9,910	8.9	6,890	7.3	2,667	7.7	4,223	7.2
Living area												
Large city	67,749	33.7	31,940	35.4	35,809	32.2	30,958	33.0	12,329	35.5	18,629	31.6
Medium-sized city	85,423	42.4	37,856	42.0	47,567	42.8	39,508	42.1	14,409	41.5	25,099	42.5
Small city/village	48,091	23.9	20,304	22.5	27,787	25.0	23,285	24.8	7,985	23.0	15,300	25.9
Partnership status												
Married/cohabiting	128,644	63.9	50,876	56.5	77,768	70.0	59,516	63.5	17,417	50.2	42,099	71.3
Single	72,619	36.1	39,224	43.5	33,395	30.0	34,235	36.5	17,306	49.8	16,929	28.7
Branch of industry												
Services	57,947	28.8	21,950	24.4	35,997	32.4	33,733	36.0	11,494	33.1	22,239	37.7
Manufacturing	22,886	11.4	4,578	5.1	18,308	16.5	15,729	16.8	3,461	10.0	12,268	20.8
Hospitality	11,608	5.8	5,476	6.1	6,132	5.5	5,433	5.8	2,205	6.4	3,228	5.5
Transports	8,683	4.3	913	1.0	7,770	7.0	3,791	4.0	421	1.2	3,370	5.7
Construction/installation	11,244	5.6	1,220	1.4	10,024	9.0	4,070	4.3	558	1.6	3,512	5.9
Care and education	49,393	24.5	35,705	39.6	13,688	12.3	14,451	15.4	9,015	26.0	5,436	9.2
Missing information	39,502	19.6	20,258	22.5	19,244	17.3	16,544	17.6	7,569	21.8	8,975	15.2
SA during the exposure interval (2010–2014)												
No	156,408	77.7	66,687	74.0	89,721	80.7	90,878	96.9	33,609	96.8	57,269	97.0
Yes (in any diagnoses)	44,855	22.3	23,413	26.0	21,442	19.3	2,873	3.1	1,114	3.2	1,759	3.0
Only mental diagnoses	3,405	1.7	2,312	2.6	1,093	1.0	69	0.1	39	0.1	30	0.1
Only somatic diagnoses^c^	39,381	19.6	19,579	21.7	19,802	17.8	2,781	3.0	1,062	3.1	1,719	2.9
Mental and somatic diagnoses	2,069	1.0	1,522	1.7	547	0.5	23	0	13	0	10	0
DP during the exposure interval (2010–2014)												
No	184,540	91.7	80,265	89.1	104,275	93.8	93,751	100	34,723	100	59,028	100
Yes (in any diagnoses)	16,723	8.3	9,835	10.9	6,888	6.2						
Only mental diagnoses	3,193	1.6	2,085	2.3	1,108	1.0						
Only somatic diagnoses^d^	13,520	6.7	7,741	8.6	5,779	5.2						
Mental and somatic diagnoses	< 10	0	< 10	0	< 10	0						
SA and/or DP during the exposure interval (2010–2014)^e^												
No	143,988	71.5	59,742	66.3	84,246	75.8	90,878	96.9	33,609	96.8	57,269	97.0
Yes (in any diagnoses)	57,275	28.5	30,358	33.7	26,917	24.2	2,873	3.1	1,114	3.2	1,759	3.0
Number of net days^f^												
>0–30	11,171	5.6	5,994	6.7	5,177	4.7	652	0.7	286	0.8	366	0.6
>30–90	16,694	8.3	8,431	9.4	8,263	7.4	1,219	1.3	495	1.4	724	1.2
>90–180	8,903	4.4	4,505	5.0	4,398	4.0	668	0.7	245	0.7	423	0.7
>180–365 (>180 for cohort70)	8,040	4.0	4,212	4.7	3,828	3.4	334	0.4	88	0.3	246	0.4
>365–731	7,585	3.8	4,402	4.9	3,183	2.9						
>731	4,882	2.4	2,814	3.1	2,068	1.9						
Only with mental diagnoses	5,580	2.8	3,643	4.0	1,937	1.7	69	0.1	39	0.1	30	0.1
Number of net days^f^												
>0–30	965	0.5	714	0.8	251	0.2	22	0	>12	0	<10	0
>30–90	1,316	0.7	845	0.9	471	0.4	27	0	16	0	11	0
>90–180	736	0.4	480	0.5	256	0.2	12	0	<10	0	<10	0
>180–365 (>180 for cohort70)	890	0.4	570	0.6	320	0.3	<10	0	<10	0	<10	0
>365–731	954	0.5	613	0.7	341	0.3						
>731	719	0.4	421	0.5	298	0.3						
Only with somatic diagnoses	48,936	24.3	24,681	27.4	24,255	21.8	2,781	3.0	1,062	3.1	1,719	2.9
Number of net days^f^												
>0–30	10,188	5.1	5,263	5.8	4,925	4.4	630	0.7	272	0.8	358	0.6
>30–90	14,828	7.4	7,187	8.0	7,641	6.9	1,183	1.3	475	1.4	708	1.2
>90–180	7,604	3.8	3,623	4.0	3,981	3.6	646	0.7	234	0.7	412	0.7
>180–365 (>180 for cohort70)	6,538	3.2	3,184	3.5	3,354	3.0	322	0.3	81	0.2	241	0.4
>365–731	6,019	3.0	3,327	3.7	2,692	2.4						
>731	3,759	1.9	2,097	2.3	1,662	1.5						
In paid work during follow-up (i.e., income above our limit to be classified as being in paid work)												
No	40,513	20.1	20,014	22.2	20,499	18.4	16,058	17.1	6,283	18.1	9,775	16.6
Yes	160,750	79.9	70,086	77.8	90,664	81.6	77,693	82.9	28,440	81.9	49,253	83.4
in 2015:	151,698	75.4	66,397	73.7	85,301	76.7	71,663	76.4	26,325	75.8	45,338	76.8
in 2016:	125,325	62.3	54,446	60.4	70,879	63.8	61,305	65.4	22,487	64.8	38,818	65.8
in 2017:	106,405	52.9	45,738	50.8	60,667	54.6	53,131	56.7	19,457	56.0	33,674	57.0
in 2018:	93,702	46.6	40,024	44.4	53,678	48.3	46,120	49.2	16,833	48.5	29,287	49.6
Throughout 2015–2018:	81,406	40.4	35,108	39.0	46,298	41.6	39,257	41.9	14,528	41.8	24,729	41.9

^a^Including individuals with missing information on educational level (n = 351; 0.2% in cohort65 and n = 287; 0.3% in cohort70), ^b^Including individuals with missing information on birth country (n < 10), ^c^Individuals who had SA in missing diagnoses (n = 640; 0.3% in cohort65 and n = 64; 0.07% in cohort70) were coded as having SA in somatic diagnoses,^d^Individuals who had DP in missing diagnoses in cohort65 (n = 955; 0.5%) were coded as having DP in somatic diagnoses (DP was not possible for cohort70 due to age regulations), ^e^Exposure variable in the main analyses, ^f^Total number of net days during the exposure interval (2010–2014)

### Association between prior SADP and time until work exit

**Fig. 2:**
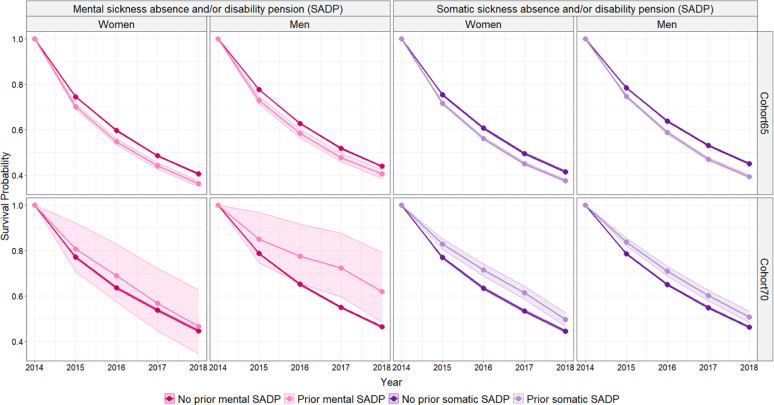
Unadjusted survival probability of remaining in paid work, stratified by prior SADP status. *Note* The shaded areas around the curves represent 95% confidence intervals

Figure 2 illustrates the unadjusted survival probability of remaining in paid work from baseline in 2014 until the end of follow-up in 2018, stratified by prior SADP status.

#### Mental SADP

Results for the associations between mental SADP and time until work exit are presented in Table 2. When adjusted for sociodemographic factors and branch of industry, significant associations were found only among women in cohort65 (Table 2, Model 2). Among these women, prior mental SADP (yes/no) was associated with a slightly shorter time until work exit (TR 0.94; 95% CI 0.93–0.96) compared to women without mental SADP. However, the direction of the association differed depending on the number of SADP days; women with > 30–90 days had shorter time until work exit (0.93; 0.89–0.98) while women with > 731 days had longer time until exit (1.13; 1.05–1.21).

**Table 2:** Time ratios for mental sickness absence and/or disability pension (SADP) and time until work exit

	All in cohort65^a^			Women in cohort65^a^			Men in cohort65^a^		
	Crude TR (95% CI)	Model 1	Model 2	Crude TR (95% CI)	Model 1	Model 2	Crude TR (95% CI)	Model 1	Model 2
Mental SADP (ref: no mental SADP)^b^									
Yes (any mental SADP)^c^	**0.90 (0.89–0.92)**	**0.93 (0.91–0.94)**	**0.96 (0.95–0.98)**	**0.91 (0.89–0.93)**	**0.92 (0.90–0.94)**	**0.94 (0.93–0.96)**	**0.92 (0.89–0.95)**	**0.93 (0.90–0.96)**	1.00 (0.97–1.03)
Number of days									
> 0–30	0.94 (0.90–1.00)	0.98 (0.93–1.03)	0.96 (0.92–1.01)	0.96 (0.90–1.02)	0.97 (0.91–1.03)	0.96 (0.91–1.01)	0.98 (0.88–1.09)	1.00 (0.90–1.11)	0.98 (0.89–1.08)
> 30–90	**0.94 (0.90–0.99)**	0.96 (0.91–1.00)	0.96 (0.93–1.00)	**0.93 (0.88–0.99)**	**0.93 (0.88–0.98)**	**0.93 (0.89–0.98)**	1.00 (0.93–1.08)	1.00 (0.92–1.08)	1.03 (0.96–1.10)
> 90–180	**0.92 (0.87–0.98)**	**0.91 (0.86–0.97)**	0.96 (0.91–1.01)	0.96 (0.89–1.03)	0.93 (0.86–1.00)	0.94 (0.88–1.00)	**0.90 (0.81–0.99)**	**0.88 (0.79–0.97)**	1.00 (0.91–1.10)
> 180–365	0.95 (0.90–1.00)	**0.92 (0.87–0.98)**	0.96 (0.92–1.01)	0.96 (0.90–1.03)	0.93 (0.87–1.00)	0.96 (0.90–1.02)	0.95 (0.87–1.05)	0.91 (0.83–1.00)	0.96 (0.88–1.04)
> 365–731	0.95 (0.90–1.00)	0.96 (0.91–1.02)	1.02 (0.97–1.07)	0.98 (0.91–1.05)	0.98 (0.91–1.04)	1.01 (0.95–1.07)	0.93 (0.85–1.02)	0.93 (0.85–1.02)	1.04 (0.96–1.13)
> 731	0.95 (0.89–1.01)	0.99 (0.93–1.06)	**1.12 (1.06–1.18)**	0.97 (0.89–1.05)	1.00 (0.92–1.09)	**1.13 (1.05–1.21)**	0.95 (0.86–1.04)	0.98 (0.89–1.09)	1.10 (1.00–1.20)

	All in cohort70^e^			Women in cohort70^e^			Men in cohort70^e^		
	Crude TR (95% CI)	Model 1	Model 2	Crude TR (95% CI)	Model 1	Model 2	Crude TR (95% CI)	Model 1	Model 2
Mental SADP (ref: no mental SADP)^b^									
Yes (any mental SADP)^c^	1.18 (0.97–1.44)	1.15 (0.94–1.39)	1.07 (0.88–1.30)	1.06 (0.83–1.36)	1.02 (0.80–1.31)	0.94 (0.74–1.20)	1.39 (1.00–1.93)	1.35 (0.97–1.87)	1.30 (0.95–1.79)
Number of days									
> 0–30	1.30 (0.85–1.99)	1.27 (0.83–1.94)	1.13 (0.75–1.71)	1.16 (0.71–1.89)	1.11 (0.68–1.80)	0.99 (0.62–1.58)	1.78 (0.72–4.38)	1.75 (0.71–4.30)	1.60 (0.67–3.80)
> 30–90	1.31 (0.89–1.93)	1.27 (0.86–1.86)	1.21 (0.84–1.76)	1.26 (0.78–2.06)	1.21 (0.74–1.96)	1.06 (0.66–1.71)	1.41 (0.75–2.68)	1.37 (0.72–2.58)	1.50 (0.81–2.77)
> 90–180	1.35 (0.76–2.39)	1.32 (0.74–2.33)	1.26 (0.72–2.19)	2.32 (0.64–8.44)	2.25 (0.62–8.14)	2.28 (0.66–7.94)	1.05 (0.56–1.99)	1.03 (0.55–1.94)	0.91 (0.49–1.68)
> 180	0.83 (0.47–1.47)	0.80 (0.45–1.42)	0.74 (0.43–1.29)	0.77 (0.36–1.61)	0.71 (0.34–1.50)	0.69 (0.34–1.43)	0.93 (0.38–2.28)	0.92 (0.37–2.25)	0.80 (0.34–1.91)

^a^cohort65 = aged 65–69 in 2014, ^b^Exposure variable, ^c^Binary variable (yes/no), ^d^cohort70 = aged ≥ 70 in 2014. TR = Time Ratio. 95% CI = 95% Confidence Interval. Significant associations are in bold. TRs below 1 indicate shorter time until work exit, and TRs above 1 longer time until work exit. Model 1 was adjusted for age, sex, educational level, birth country, living area, and partnership status. Model 2 was adjusted for the same variables as Model 1 and branch of industry. The estimates for covariates were obtained from the model in which SADP was categorised by the number of days and were almost identical to those from the model where SADP was measured as yes/no. For mental SADP, the scale parameter was 0.58 for women and 0.57 for men in cohort65, and 0.63 for both women and men in cohort70

#### Somatic SADP

Results for the associations between somatic SADP and time until work exit are shown in Table 3. In cohort65, women with prior somatic SADP had a slightly shorter time until work exit (TR 0.95; 0.94–0.96) than women in the same cohort without somatic SADP, when adjusted for sociodemographic factors and branch of industry (see Table 3, Model 2). A similar result was observed among men in cohort65 (0.94; 0.93–0.95) (see Table 3, Model 2). For both women and men in cohort65, the strength of the association was similar regardless of the number of somatic SADP days, except for the highest category (> 731 days), which showed a non-significant association (see Table 3, Model 2).

**Table 3:** Time ratios for somatic sickness absence and/or disability pension (SADP) and time until work exit

	All in cohort65^a^			Women in cohort65^a^			Men in cohort65^a^		
	Crude TR (95% CI)	Model 1	Model 2	Crude TR (95% CI)	Model 1	Model 2	Crude TR (95% CI)	Model 1	Model 2
Somatic SADP (ref: no somatic SADP)^b^									
Yes (any somatic SADP)^c^	**0.90 (0.89–0.91)**	**0.93 (0.92–0.94)**	**0.94 (0.94–0.95)**	**0.92 (0.91–0.93)**	**0.94 (0.93–0.95)**	**0.95 (0.94–0.96)**	**0.89 (0.88–0.90)**	**0.92 (0.91–0.93)**	**0.94 (0.93–0.95)**
Number of days									
> 0–30	**0.91 (0.89–0.92)**	**0.94 (0.93–0.96)**	**0.94 (0.92–0.95)**	**0.93 (0.91–0.95)**	**0.95 (0.93–0.98)**	**0.95 (0.93–0.96)**	**0.90 (0.88–0.92)**	**0.92 (0.90–0.95)**	**0.93 (0.91–0.95)**
> 30–90	**0.92 (0.91–0.93)**	**0.95 (0.94–0.96)**	**0.94 (0.93–0.95)**	**0.94 (0.92–0.96)**	**0.96 (0.95–0.98)**	**0.95 (0.93–0.97)**	**0.91 (0.89–0.92)**	**0.93 (0.91–0.95)**	**0.93 (0.91–0.94)**
> 90–180	**0.91 (0.89–0.92)**	**0.93 (0.91–0.95)**	**0.94 (0.92–0.95)**	**0.91 (0.89–0.94)**	**0.93 (0.90–0.95)**	**0.93 (0.91–0.95)**	**0.91 (0.88–0.93)**	**0.93 (0.91–0.96)**	**0.95 (0.92–0.97)**
> 180–365	**0.90 (0.88–0.92)**	**0.92 (0.90–0.94)**	**0.93 (0.91–0.95)**	**0.93 (0.91–0.96)**	**0.94 (0.91–0.97)**	**0.94 (0.92–0.97)**	**0.88 (0.85–0.90)**	**0.89 (0.87–0.92)**	**0.92 (0.90–0.94)**
> 365–731	**0.87 (0.86–0.89)**	**0.91 (0.89–0.93)**	**0.96 (0.94–0.98)**	**0.88 (0.86–0.91)**	**0.92 (0.89–0.94)**	**0.96 (0.94–0.99)**	**0.88 (0.85–0.91)**	**0.91 (0.88–0.94)**	**0.96 (0.93–0.99)**
> 731	**0.84 (0.81–0.86)**	**0.91 (0.89–0.93)**	0.99 (0.97–1.02)	**0.84 (0.81–0.87)**	**0.92 (0.89–0.95)**	1.00 (0.97–1.04)	**0.84 (0.81–0.88)**	**0.90 (0.87–0.94)**	0.98 (0.95–1.01)

	All in cohort70^d^			Women in cohort70^d^			Men in cohort70^d^		
	Crude TR (95% CI)	Model 1	Model 2	Crude TR (95% CI)	Model 1	Model 2	Crude TR (95% CI)	Model 1	Model 2
Somatic SADP (ref: no somatic SADP)^b^									
Yes (any somatic SADP)^c^	**1.12 (1.08–1.16)**	**1.10 (1.06–1.14)**	**1.05 (1.01–1.08)**	**1.14 (1.07–1.20)**	**1.11 (1.05–1.17)**	1.05 (0.99–1.11)	**1.11 (1.06–1.16)**	**1.09 (1.04–1.14)**	1.04 (1.00–1.09)
Number of days									
> 0–30	**1.16 (1.08–1.25)**	**1.13 (1.05–1.22)**	1.07 (1.00–1.15)	**1.21 (1.09–1.36)**	**1.18 (1.06–1.32)**	**1.13 (1.01–1.26)**	**1.12 (1.01–1.23)**	1.09 (0.99–1.20)	1.03 (0.94–1.13)
> 30–90	**1.13 (1.07–1.19)**	**1.11 (1.05–1.17)**	1.04 (0.99–1.10)	**1.11 (1.03–1.21)**	1.09 (1.00–1.19)	1.03 (0.95–1.11)	**1.14 (1.06–1.23)**	**1.12 (1.04–1.20)**	1.05 (0.98–1.13)
> 90–180	**1.13 (1.05–1.21)**	**1.11 (1.03–1.20)**	1.08 (1.00–1.15)	**1.17 (1.04–1.33)**	**1.15 (1.02–1.30)**	1.08 (0.96–1.21)	**1.10 (1.01–1.21)**	1.09 (1.00–1.19)	1.07 (0.98–1.17)
> 180	0.99 (0.89–1.09)	0.97 (0.88–1.07)	0.95 (0.86–1.05)	0.91 (0.75–1.11)	0.89 (0.73–1.08)	0.86 (0.72–1.04)	1.01 (0.90–1.13)	1.00 (0.89–1.12)	0.99 (0.88–1.10)

^a^cohort65 = aged 65–69 in 2014, ^b^Exposure variable, ^c^Binary variable (yes/no), ^d^cohort70 = aged ≥ 70 in 2014. TR = Time Ratio. 95% CI = 95% Confidence Interval. Significant associations are in bold. TRs below 1 indicate shorter time until work exit, and TRs above 1 longer time until work exit. Model 1 was adjusted for age, sex, educational level, birth country, living area, and partnership status. Model 2 was adjusted for the same variables as Model 1 and branch of industry. The estimates for covariates were obtained from the model in which SADP was categorised by the number of days and were almost identical to those from the model where SADP was measured as yes/no. For somatic SADP, the scale parameter was 0.58 for women and 0.57 for men in cohort65, and 0.64 for women and 0.63 for men in cohort70

In cohort70, most associations were insignificant when the analyses were adjusted for sociodemographic factors and branch of industry (see Table 3, Model 2). However, women with > 0–30 somatic SA days had a slightly longer time until work exit (1.13; 1.01–1.23) compared to women without somatic SA. This was not observed among men in cohort70. Results for somatic SADP were very similar to those for all-cause SADP; the latter are presented in Supplementary Table 1.

### Covariates

Time Ratios for covariates are presented in Supplementary Table 1 (see Model 2). Women had marginally shorter time until work exit than men in cohort65 (TR 0.98; 0.97–0.99), but not in in cohort70. Elementary and high school education were both associated with shorter time until work exit, compared to university education, among women and men in cohort65 (TRs with 95%CI varied between 0.88 and 0.96) and men in cohort70 (TRs with 95%CI varied between 0.91 and 0.97), but not among women in cohort70.

In both cohorts and sexes, shorter time until work exit was observed among people who worked in manufacturing (TRs with 95% CIs varied between 0.74 and 0.89) and in unknown branch of industry (TRs with 95%CI varied between 0.36 and 0.41 for cohort65, and between 0.50 and 0.59 for cohort70). Conversely, longer time until work exit was observed in both cohorts and sexes among individuals who worked in care/education (TRs with 95%CI varied between 1.03 and 1.12) and transport (TRs with 95%CI varied between 1.11 and 1.25 for cohort65, and between 1.05 and 1.29 for cohort70). The two other studied industries, construction/installation and hospitality, showed more nuanced results depending on cohort and sex.

## Discussion

This prospective population-based cohort study aimed to explore if prior sickness absence (> 14 days) and/or disability pension (SADP) in mental and/or somatic diagnoses were associated with time until work exit after ages 65–69 and ≥ 70, respectively, among women and men. All the included individuals were actively engaged in the labour market, at least to some degree, at baseline in 2014 when they were aged 65–69 and ≥ 70, respectively. The results showed that ~ 80% remained in paid work for at least some time during the 4-year follow-up in both cohorts and sexes, and that ~ 41% worked throughout the follow-up. Neither mental nor somatic SADP was strongly associated with time until work exit.

The weak association between prior SADP and time until work exit could depend on many factors. SADP might not play a big role for work exit among individuals who already are in paid work after the standard retirement age, which was the case for our study cohorts. Instead, other factors might play a bigger role for remaining in paid work after age 65 and 70. For example, some people might extend their working life involuntarily, for example due to financial reasons [54, 55]. Working for financial reasons has been found to be more common among people who have both low level of income and low level of education [20] and among individuals with worse self-rated mental health [56]. Other factors that could motivate people to remain in paid work could be stimulating and interesting work [57], strong work motivation and commitment [54], wishing to maintain daily routines [55], to feel appreciated by others [58], or having a partner who is in paid work [59, 60].

Another possible reason for the weak association between SA and work exit might be that SA could in fact improve one’s ability to remain in paid work despite a temporary or chronic condition, for example by making it possible to allocate time to rehabilitation and recovery. One of the purposes of SA benefit policies is indeed to avoid permanent exit from the labour market [61, 62]. It has been suggested that individuals with high levels of SA might have later permanent exits from labour market, as the SA enables them to remain in the labour market to some extent in spite of partial or on-and-off work incapacity [63].

While the association between SADP and time until work exit was weak, it is important to remember that SA and DP only capture the morbidities that affect individuals’ work capacity. That is, individuals in our study cohorts could have had health issues that did not affect their working capacity. Also, it is important to note that the individuals included in this study were actively engaged in the labour market at baseline, representing ~ 6% (cohort70) and ~ 27% (cohort65) of the population-based source cohorts. It is unlikely that the study cohorts included individuals with poorest health, who are likely to leave the labour market well before age 65 [32–36]. Consequently, our study cohorts could be selected in many ways, such as regarding health status, work capacity, or other factors that facilitate remaining in paid work, such as flexible work arrangements, adapted working hours or work tasks.

Our study examined new and important aspects that have not been studied before, to the best of our knowledge. One unique aspect was studying individuals aged ≥ 70 separately. Another aspect was investigating the length of prior SADP rather than only using a binary measure (yes/no). For somatic SADP, time until work exit was largely the same regardless of the number of SADP days, suggesting that it might be somatic SADP itself rather than the number of number of days that is of importance for work exit. For mental SADP, the direction of the associations differed by length of mental SADP in cohort65, as women with > 30–90 days had shorter time and women with > 731 days longer time until work exit. The latter result can seem counterintuitive and should be investigated in more detail in future studies. One possible contributing factor could be that women with long SADP may need to compensate for financial losses by remaining in paid work. This pattern has been observed in the UK among women whose careers were characterized by interruptions and predominantly part-time work [15]. However, the likelihood of extending working life is generally higher among people with stable labour market histories than those with unstable labour market histories [64, 65].

A third new aspect of this study was that we also considered branch of industry when exploring the association between SADP and work exit. We found that individuals who worked in care and education and in transport had longer time until work exit compared to people who worked in the service branch. It is likely that working conditions, including work demands, vary greatly between and within different branches of industries, which can affect individuals’ possibilities to extend their working lives. It is possible that working conditions become even more important with higher age, as older people are more likely to develop different types of morbidities [27]. Differences in working conditions and work demands could also affect the need of SA. For example, a bone fracture might impair the work capacity of a construction worker but have little or no impact on an office worker.

Results of this study partly align with the results from the few previous studies in the field. Like two prior Swedish studies [12, 14], this study found an association between prior SADP and future participation in paid work. However, the associations were weaker in this study than in the abovementioned studies. Furthermore, we did not observe major differences between mental and somatic SADP, in contrast to one previous Swedish study [12]. In that study, individuals with prior mental SA had lower odds of being in paid work after age 65 than people with prior somatic SA, while individuals with prior DP had low odds regardless of the DP diagnosis. The differences between the present and the two prior studies could depend on differences in study designs. For example, the present study examined another birth cohort, applied more strict inclusion criteria, and studied 65–69-year-olds and ≥ 70-year-olds separately (instead of examining all aged > 65). Also, our outcome was time until work exit, whereas the two prior studies used a binary measure for being in paid work (yes/no).

When it comes to sex differences, both our study and prior studies from Sweden [12, 14], Finland [13] and UK [15] show that women are less likely to extend their working life beyond standard retirement age compared to men [12–15]. However, in our current study, the difference was only marginal in cohort65 and became nonsignificant in cohort70 after adjusting for branch of industry. This suggests that when type of work is considered, sex differences can level off with increased age.

Future research could expand the knowledge generated by this study by having longer exposure and follow-up intervals, and stratifying analyses by income. It would also be good to consider prior labour market engagement in Sweden as this could affect the amount of pension a person gets, and thus affect financial incentives to remain in paid work. In the present study, all individuals had lived in Sweden all five years prior the baseline and were engaged in the labour market to some degree at baseline. Further, it is important to examine differences regarding sex, branch of industry, and different employment sectors in more detail. Future studies could also investigate whether specific morbidities are associated with earlier work exit among those who continue working until and beyond ages 65 and 70. It is important to keep in mind that transition towards work exit is a complex, individual process that is affected by many factors that can accumulate over decades.

### Strengths and limitations

As every study, the present study has strengths and limitations. Strengths include the longitudinal prospective cohort design, covering nine years in total, and the use of microdata linked at the individual level from three different nationwide registers of high quality [66]. All individuals in the whole country who fulfilled the inclusion criteria were included, and the large cohorts made sub-group analyses possible. As the data was administrative, there was no risk of recall bias, and there were no drop outs and no loss to follow-up. An important knowledge gap was filled by studying those aged ≥ 70 separately from those aged 65–69, by analysing SADP as numbers of days in addition to binary measures, and by considering branch of industry in the analyses. When it comes to the generalizability of the results, it is important to note that both SADP and paid work after the standard retirement age are highly context-dependent. The results from this study could be generalized to other countries with similar pension and sickness absence insurance systems but not to post-retirement employment in general.

Limitations of this study, in turn, include that we were only able to measure work exits on a yearly basis, which made the outcome measure quite crude. For example, if a person exited from paid work at the beginning of 2016 but fulfilled the annual income limit during the month(s) which they worked, they would have been considered to have the work exit first in 2017. Also, the income limit used in this study was rather low and thus inclusive (799–856 EUR/year depending on the follow-up year), which can be seen both as a strength and a limitation. This inclusivity makes it more challenging to detect whether the studied associations could have differed depending on the income level. It is also possible that some people substantially decreased their labour market participation but still fulfilled our income limit and were thus considered to be in paid work. However, the inclusivity ensures that individuals with low salaries and/or those who worked part-time were not excluded from the study, which was important as our focus was to examine those who were active in the labour market, at least to some degree. Another limitation is that we did not have data on the exact work-related factors, such as psychosocial and physical working conditions, which could influence both SADP and exit from paid work.

## Conclusions

This prospective population-based cohort study in Sweden examined the individuals who were in paid work when aged 65–69 and ≥ 70, respectively. Most individuals (~ 80%) remained in paid work for at least some time, and ~ 41% worked throughout the 4-year follow-up. History of mental and somatic SADP in the five prior years were only weakly, or not at all, associated with time until work exit. This result implies that history of reduced work capacity, as indicated by SADP, is not necessarily a determinant of work exit at higher ages, in contrast to existing literature on younger cohorts. Thus, our findings underscore the importance of focusing on post-retirement age workers to understand determinants of extended working life. These insights are crucial for older workers, employers, policymakers, and insurers to enable extended working lives and address challenges related to an aging workforce.

## Supplementary Information

Below is the link to the electronic supplementary material.

Supplementary Material 1.

## Data Availability

The data used in this study is administered by the Division of Insurance Medicine, Karolinska Institutet, and cannot be made public. According to the General Data Protection Regulation, the Swedish law SFS 2018:218, the Swedish Data Protection Act, the Swedish Ethical Review Act, and the Public Access to Information and Secrecy Act, these types of sensitive and confidential data can only be made available, after legal review, for researchers who meet the criteria for access. Readers may contact Professor Ellenor Mittendorfer-Rutz (ellenor.mittendorfer-rutz@ki.se) regarding the data.

## List of abbreviations

CI: Confidence interval
DP: Disability pension
TR: Time ratio
ICD-10: International Classification of Diseases 10th Revision
LISA: Longitudinal Integrated Database for Health Insurance and Labour Market Studies Registers
MiDAS: Micro Data for the Analysis of Social Insurance
SA: Sickness absence
SADP: Sickness absence and/or disability pension
