# Supplementary material for: Time until exit from paid work after ages 65–69 and ≥ 70, respectively: importance of prior sickness absence and disability pension
Source: BMC Public Health. 2025 Sep 9;25:3040. doi: 10.1186/s12889-025-24425-1 (PMC12418626; doi:10.1186/s12889-025-24425-1)
Supplement: Supplementary file 1 — Supplementary Material 1. [file 12889_2025_24425_MOESM1_ESM.pdf]

Supplementary Material 1

Time until exit from paid work after ages 65-69 and ≥70, respectively: importance of prior sickness absence and disability pension

Aleksiina Martikainen[1], Kristina Alexanderson[1], Pia Svedberg[1] & Kristin Farrants[1]

[1]Division of Insurance Medicine, Department of Clinical Neuroscience, Karolinska Institutet, SE-171 77 Stockholm, Sweden

Supplementary Table 1

Time Ratios for All-Cause Sickness Absence and/or Disability Pension (SADP) and Time Until Work Exit

	All in cohort65[a]			Women in cohort65[a]			Men in cohort65[a]		
	**Crude TR (95% CI)**	**Model 1**	**Model 2**	**Crude TR (95% CI)**	**Model 1**	**Model 2**	**Crude TR (95% CI)**	**Model 1**	**Model 2**
**All-cause SADP (ref: no SADP)[b]**									
Yes (any SADP)[c]	**0.90 (0.89–0.91)**	**0.93 (0.92–0.94)**	**0.95 (0.94–0.95)**	**0.92 (0.91–0.93)**	**0.94 (0.93–0.95)**	**0.95 (0.94–0.96)**	**0.89 (0.88–0.90)**	**0.92 (0.91–0.93)**	**0.94 (0.93–0.95)**
Number of days									
>0-30	**0.91 (0.90–0.93)**	**0.94 (0.93–0.96)**	**0.94 (0.92–0.95)**	**0.93 (0.91–0.96)**	**0.96 (0.94–0.98)**	**0.95 (0.93–0.97)**	**0.90 (0.88–0.92)**	**0.93 (0.91–0.95)**	**0.93 (0.91–0.95)**
>30-90	**0.92 (0.91–0.93)**	**0.95 (0.94–0.96)**	**0.94 (0.93–0.95)**	**0.94 (0.92–0.96)**	**0.96 (0.94–0.98)**	**0.95 (0.93–0.96)**	**0.91 (0.89–0.93)**	**0.94 (0.92–0.95)**	**0.93 (0.92–0.95)**
>90-180	**0.91 (0.89–0.92)**	**0.93 (0.91–0.95)**	**0.94 (0.93–0.96)**	**0.92 (0.90–0.94)**	**0.93 (0.91–0.96)**	**0.94 (0.92–0.96)**	**0.91 (0.88–0.93)**	**0.93 (0.90–0.95)**	**0.95 (0.93–0.97)**
>180-365	**0.90 (0.88–0.91)**	**0.91 (0.89–0.93)**	**0.93 (0.91–0.95)**	**0.92 (0.90–0.95)**	**0.92 (0.90–0.95)**	**0.93 (0.91–0.96)**	**0.88 (0.86–0.90)**	**0.89 (0.87–0.92)**	**0.93 (0.90–0.95)**
>365-731	**0.87 (0.86–0.89)**	**0.91 (0.89–0.93)**	**0.96 (0.94–0.98)**	**0.89 (0.86–0.91)**	**0.91 (0.89–0.94)**	**0.96 (0.94–0.98)**	**0.88 (0.85–0.90)**	**0.90 (0.88–0.93)**	**0.96 (0.94–0.99)**
>731	**0.83 (0.81–0.85)**	**0.90 (0.88–0.93)**	1.00 (0.98–1.02)	**0.84 (0.82–0.87)**	**0.91 (0.88–0.94)**	1.01 (0.98–1.03)	**0.84 (0.81–0.88)**	**0.90 (0.87–0.94)**	0.99 (0.96–1.02)
**Age[d]**	**1.06 (1.06–1.07)**	**1.06 (1.06–1.06)**	**1.07 (1.06–1.07)**	**1.07 (1.07–1.08)**	**1.07 (1.07–1.07)**	**1.08 (1.08–1.08)**	**1.06 (1.05–1.06)**	**1.06 (1.05–1.06)**	**1.06 (1.05–1.06)**
**Sex (ref: men)**									
Women	**0.93 (0.93–0.94)**	**0.94 (0.93–0.95)**	**0.98 (0.97–0.99)**						
**Educational level (ref: university/college)**									
Elementary	**0.90 (0.89–0.91)**	**0.89 (0.88–0.90)**	**0.90 (0.89–0.91)**	**0.87 (0.85–0.88)**	**0.86 (0.84–0.87)**	**0.92 (0.90–0.93)**	**0.90 (0.89–0.91)**	**0.91 (0.90–0.92)**	**0.89 (0.88–0.90)**
High school	**0.90 (0.89–0.91)**	**0.90 (0.89–0.91)**	**0.93 (0.92–0.93)**	**0.91 (0.90–0.92)**	**0.91 (0.90–0.92)**	**0.95 (0.94–0.96)**	**0.89 (0.88–0.90)**	**0.90 (0.89–0.91)**	**0.91 (0.90–0.92)**
**Birth country (ref: Sweden)**									
Other	1.01 (1.00–1.03)	**1.03 (1.01–1.04)**	1.01 (1.00–1.02)	**1.03 (1.01–1.05)**	**1.04 (1.02–1.06)**	**1.02 (1.01–1.04)**	1.01 (0.99–1.03)	1.01 (1.00–1.03)	1.00 (0.98–1.02)
**Living area (ref: big city)**									
Medium-size city	**0.96 (0.95–0.97)**	**0.98 (0.97–0.99)**	**1.03 (1.02–1.04)**	**0.97 (0.96–0.99)**	1.00 (0.98–1.01)	**1.05 (1.04–1.06)**	**0.95 (0.94–0.96)**	**0.96 (0.95–0.98)**	1.01 (1.00–1.02)
Rural area	**0.98 (0.97–0.99)**	1.00 (0.99–1.01)	**1.07 (1.06–1.08)**	0.98 (0.97–1.00)	1.01 (1.00–1.03)	**1.08 (1.07–1.10)**	**0.97 (0.96–0.98)**	1.00 (0.98–1.01)	**1.07 (1.06–1.08)**
**Partnership status (ref: married/cohabiting)**									
Single	**0.98 (0.97–0.98)**	0.99 (0.98–1.00)	**0.97 (0.96–0.97)**	**1.02 (1.01–1.03)**	**1.02 (1.01–1.03)**	**0.98 (0.97–0.99)**	**0.95 (0.94–0.96)**	**0.96 (0.95–0.97)**	**0.96 (0.95–0.97)**
**Branch of industry (ref: services)**									
Manufacturing	**0.86 (0.85–0.87)**		**0.87 (0.86–0.88)**	**0.82 (0.80–0.84)**		**0.82 (0.80–0.84)**	**0.86 (0.84–0.87)**		**0.88 (0.87–0.89)**
Hospitality	0.99 (0.98–1.01)		1.01 (1.00–1.03)	1.01 (0.99–1.03)		1.02 (1.00–1.05)	0.99 (0.97–1.01)		1.01 (0.99–1.04)
Transport	**1.14 (1.11–1.16)**		**1.16 (1.14–1.18)**	**1.17 (1.10–1.24)**		**1.18 (1.11–1.25)**	**1.11 (1.08–1.13)**		**1.15 (1.12–1.17)**
Construction/ installation	0.99 (0.97–1.01)		1.02 (1.00–1.03)	**1.12 (1.06–1.18)**		**1.12 (1.06–1.17)**	**0.95 (0.94–0.97)**		1.00 (0.98–1.02)
Care/education	**1.05 (1.04–1.06)**		**1.07 (1.06–1.08)**	**1.06 (1.05–1.08)**		**1.08 (1.06–1.09)**	**1.12 (1.10–1.14)**		**1.10 (1.08–1.12)**
Unknown	**0.38 (0.37–0.38)**		**0.38 (0.38–0.38)**	**0.40 (0.40–0.41)**		**0.40 (0.40–0.41)**	**0.36 (0.35–0.36)**		**0.36 (0.36–0.37)**
	**All in cohort70[e]**			**Women in cohort70[e]**			**Men in cohort70[e]**		
	**Crude TR (95% CI)**	**Model 1**	**Model 2**	**Crude TR (95% CI)**	**Model 1**	**Model 2**	**Crude TR (95% CI)**	**Model 1**	**Model 2**
**All-cause SADP (ref: no SADP)[b]**									

Yes (any SADP)[c]	**1.12 (1.08–1.16)**	**1.10 (1.06–1.14)**	**1.05 (1.01–1.08)**	**1.14 (1.08–1.21)**	**1.11 (1.05–1.18)**	1.05 (0.99–1.11)	**1.11 (1.06–1.16)**	**1.09 (1.04–1.14)**	1.05 (1.00–1.09)
Number of days									
>0-30	**1.17 (1.09–1.26)**	**1.14 (1.06–1.23)**	**1.08 (1.01–1.16)**	**1.24 (1.11–1.39)**	**1.21 (1.08–1.35)**	**1.15 (1.03–1.28)**	**1.12 (1.02–1.24)**	1.10 (0.99–1.21)	1.04 (0.94–1.14)
>30-90	**1.13 (1.07–1.19)**	**1.10 (1.05–1.16)**	1.04 (0.99–1.10)	**1.11 (1.02–1.20)**	1.08 (1.00–1.17)	1.02 (0.94–1.10)	**1.14 (1.06–1.23)**	**1.12 (1.04–1.20)**	1.06 (0.99–1.13)
>90-180	**1.13 (1.06–1.22)**	**1.12 (1.04–1.20)**	**1.08 (1.01–1.16)**	**1.19 (1.05–1.34)**	**1.16 (1.03–1.31)**	1.09 (0.97–1.23)	**1.11 (1.01–1.21)**	1.09 (1.00–1.20)	1.07 (0.98–1.17)
>180	0.99 (0.90–1.09)	0.97 (0.88–1.07)	0.95 (0.86–1.04)	0.91 (0.75–1.09)	0.88 (0.73–1.06)	0.85 (0.71–1.02)	1.01 (0.90–1.14)	1.00 (0.89–1.12)	0.99 (0.88–1.10)
**Age (ref: 70-74)**									
≥75	**0.87 (0.86–0.88)**	**0.87 (0.86–0.89)**	**0.91 (0.90–0.92)**	**0.86 (0.84–0.88)**	**0.86 (0.84–0.88)**	**0.92 (0.90–0.94)**	**0.87 (0.86–0.89)**	**0.88 (0.86–0.89)**	**0.91 (0.89–0.92)**
**Sex (ref: men)**									
Women	**0.97 (0.96–0.98)**	**0.96 (0.95–0.97)**	0.99 (0.98–1.00)						
**Educational level (ref: university/college)**									
Elementary	**0.93 (0.92–0.95)**	**0.95 (0.94–0.97)**	**0.95 (0.94–0.97)**	**0.95 (0.93–0.98)**	0.98 (0.96–1.01)	0.99 (0.97–1.02)	**0.91 (0.90–0.93)**	**0.94 (0.92–0.96)**	**0.93 (0.91–0.95)**
High school	**0.96 (0.95–0.97)**	**0.97 (0.96–0.98)**	**0.98 (0.96–0.99)**	0.98 (0.96–1.00)	1.00 (0.97–1.02)	1.00 (0.98–1.02)	**0.94 (0.92–0.96)**	**0.95 (0.93–0.97)**	**0.96 (0.94–0.97)**
**Birth country (ref: Sweden)**									
Other	1.03 (1.00–1.05)	1.01 (0.99–1.03)	0.98 (0.96–1.00)	1.03 (1.00–1.07)	1.02 (0.98–1.06)	0.99 (0.96–1.02)	1.02 (1.00–1.05)	1.01 (0.98–1.03)	0.98 (0.95–1.00)
**Living area (ref: big city)**									
Medium-size city	**0.96 (0.94–0.97)**	**0.97 (0.95–0.98)**	**1.02 (1.01–1.03)**	**0.96 (0.94–0.98)**	**0.96 (0.94–0.98)**	1.03 (1.00–1.05)	**0.95 (0.94–0.97)**	**0.97 (0.95–0.98)**	1.02 (1.00–1.03)
Rural area	**0.92 (0.91–0.93)**	**0.94 (0.92–0.95)**	1.01 (1.00–1.03)	**0.92 (0.89–0.94)**	**0.93 (0.90–0.95)**	1.01 (0.99–1.04)	**0.92 (0.90–0.94)**	**0.94 (0.92–0.96)**	1.01 (1.00–1.03)
**Partnership status (ref: married/cohabiting)**									
Single	**0.98 (0.97–0.99)**	0.99 (0.98–1.00)	**0.98 (0.97–0.99)**	1.00 (0.98–1.02)	1.00 (0.98–1.02)	0.98 (0.97–1.00)	**0.97 (0.96–0.99)**	0.98 (0.96–1.00)	**0.98 (0.96–0.99)**
**Branch of industry (ref: services)**									
Manufacturing	**0.82 (0.80–0.83)**		**0.83 (0.82–0.85)**	**0.75 (0.73–0.78)**		**0.76 (0.74–0.79)**	**0.83 (0.82–0.85)**		**0.86 (0.84–0.88)**
Hospitality	1.02 (1.00–1.05)		1.03 (1.00–1.06)	1.02 (0.98–1.07)		1.02 (0.98–1.06)	1.03 (0.99–1.06)		**1.04 (1.01–1.08)**
Transport	**1.09 (1.06–1.13)**		**1.09 (1.06–1.13)**	**1.18 (1.08–1.30)**		**1.17 (1.07–1.29)**	**1.07 (1.03–1.11)**		**1.09 (1.05–1.12)**
Construction/ installation	**0.95 (0.93–0.98)**		**0.96 (0.93–0.98)**	1.06 (0.98–1.14)		1.05 (0.97–1.13)	**0.93 (0.90–0.96)**		**0.94 (0.91–0.97)**
Care/education	**1.06 (1.05–1.08)**		**1.05 (1.03–1.07)**	**1.07 (1.04–1.09)**		**1.06 (1.03–1.08)**	**1.09 (1.06–1.12)**		**1.07 (1.04–1.10)**
Unknown	**0.54 (0.53–0.54)**		**0.54 (0.53–0.55)**	**0.57 (0.56–0.59)**		**0.57 (0.56–0.59)**	**0.51 (0.50–0.52)**		**0.51 (0.50–0.52)**

Note. [a]cohort65 = aged 65-69 in 2014, [b]Exposure variable, [c]Binary variable (yes/no), [d]Measured as a continuous variable from 65 to 69, [e]cohort70 = aged ≥70 in 2014. TR = Time Ratio. 95% CI = 95% Confidence Interval. Significant associations are in bold. TRs below 1 indicate shorter time until work exit, and TRs above 1 longer time until work exit. Model 1 was adjusted for age, sex, educational level, birth country, living area, and partnership status. Model 2 was adjusted for the same variables as Model 1 and branch of industry. The estimates for covariates were obtained from the model in which SADP was categorised by the number of days and were almost identical to those from the model where SADP was measured as yes/no. For all-cause SADP, the scale parameter was 0.58 for women and 0.57 for men in cohort65, and 0.64 for women and 0.63 for men in cohort70.

Supplementary Table 2

Time Ratios for Mental Sickness Absence and/or Disability Pension (SADP) and Time Until Work Exit, with Estimates for Covariates

	All in cohort65[a]			Women in cohort65[a]			Men in cohort65[a]		
	**Crude TR (95% CI)**	**Model 1**	**Model 2**	**Crude TR (95% CI)**	**Model 1**	**Model 2**	**Crude TR (95% CI)**	**Model 1**	**Model 2**
**Mental SADP (ref: no mental SADP)[b]**									
Yes (any mental SADP)[c]	**0.90 (0.89 – 0.92)**	**0.93 (0.91 – 0.94)**	**0.96 (0.95 – 0.98)**	**0.91 (0.89 – 0.93)**	**0.92 (0.90 – 0.94)**	**0.94 (0.93 – 0.96)**	**0.92 (0.89 – 0.95)**	**0.93 (0.90 – 0.96)**	1.00 (0.97 – 1.03)
Number of days									
>0-30	0.94 (0.90 – 1.00)	0.98 (0.93 – 1.03)	0.96 (0.92 – 1.01)	0.96 (0.90 – 1.02)	0.97 (0.91 – 1.03)	0.96 (0.91 – 1.01)	0.98 (0.88 – 1.09)	1.00 (0.90 – 1.11)	0.98 (0.89 – 1.08)
>30-90	**0.94 (0.90 – 0.99)**	0.96 (0.91 – 1.00)	0.96 (0.93 – 1.00)	**0.93 (0.88 – 0.99)**	**0.93 (0.88 – 0.98)**	**0.93 (0.89 – 0.98)**	1.00 (0.93 – 1.08)	1.00 (0.92 – 1.08)	1.03 (0.96 – 1.10)
>90-180	**0.92 (0.87 – 0.98)**	**0.91 (0.86 – 0.97)**	0.96 (0.91 – 1.01)	0.96 (0.89 – 1.03)	0.93 (0.86 – 1.00)	0.94 (0.88 – 1.00)	**0.90 (0.81 – 0.99)**	**0.88 (0.79 – 0.97)**	1.00 (0.91 – 1.10)
>180-365	0.95 (0.90 – 1.00)	**0.92 (0.87 – 0.98)**	0.96 (0.92 – 1.01)	0.96 (0.90 – 1.03)	0.93 (0.87 – 1.00)	0.96 (0.90 – 1.02)	0.95 (0.87 – 1.05)	0.91 (0.83 – 1.00)	0.96 (0.88 – 1.04)
>365-731	0.95 (0.90 – 1.00)	0.96 (0.91 – 1.02)	1.02 (0.97 – 1.07)	0.98 (0.91 – 1.05)	0.98 (0.91 – 1.04)	1.01 (0.95 – 1.07)	0.93 (0.85 – 1.02)	0.93 (0.85 – 1.02)	1.04 (0.96 – 1.13)
>731	0.95 (0.89 – 1.01)	0.99 (0.93 – 1.06)	**1.12 (1.06 – 1.18)**	0.97 (0.89 – 1.05)	1.00 (0.92 – 1.09)	**1.13 (1.05 – 1.21)**	0.95 (0.86 – 1.04)	0.98 (0.89 – 1.09)	1.10 (1.00 – 1.20)
**Age[d]**	**1.06 (1.06 – 1.07)**	**1.06 (1.06 – 1.07)**	**1.07 (1.07 – 1.07)**	**1.07 (1.07 – 1.08)**	**1.07 (1.07 – 1.08)**	**1.08 (1.08 – 1.09)**	**1.06 (1.05 – 1.06)**	**1.06 (1.05 – 1.06)**	**1.06 (1.05 – 1.06)**
**Sex (ref: men)**									
Women	**0.93 (0.93 – 0.94)**	**0.94 (0.93 – 0.94)**	**0.98 (0.97 – 0.99)**						
**Educational level (ref: university/college)**									
Elementary	**0.90 (0.89 – 0.91)**	**0.88 (0.87 – 0.89)**	**0.90 (0.89 – 0.91)**	**0.87 (0.85 – 0.88)**	**0.85 (0.84 – 0.87)**	**0.91 (0.90 – 0.93)**	**0.90 (0.89 – 0.91)**	**0.90 (0.89 – 0.91)**	**0.89 (0.88 – 0.90)**
High school	**0.90 (0.89 – 0.91)**	**0.90 (0.89 – 0.91)**	**0.92 (0.91 – 0.93)**	**0.91 (0.90 – 0.92)**	**0.90 (0.89 – 0.92)**	**0.94 (0.93 – 0.95)**	**0.89 (0.88 – 0.90)**	**0.89 (0.88 – 0.90)**	**0.90 (0.89 – 0.91)**
**Birth country (ref: Sweden)**									
Other	1.01 (1.00 – 1.03)	**1.02 (1.01 – 1.04)**	1.01 (1.00 – 1.02)	**1.03 (1.01 – 1.05)**	**1.04 (1.02 – 1.06)**	**1.02 (1.01 – 1.04)**	1.01 (0.99 – 1.03)	1.01 (0.99 – 1.03)	1.00 (0.98 – 1.02)
**Living area (ref: big city)**									
Medium-size city	**0.96 (0.95 – 0.97)**	**0.98 (0.97 – 0.99)**	**1.03 (1.02 – 1.04)**	**0.97 (0.96 – 0.99)**	0.99 (0.98 – 1.01)	**1.05 (1.04 – 1.06)**	**0.95 (0.94 – 0.96)**	**0.96 (0.95 – 0.97)**	1.01 (1.00 – 1.02)
Rural area	**0.98 (0.97 – 0.99)**	1.00 (0.99 – 1.01)	**1.07 (1.06 – 1.08)**	0.98 (0.97 – 1.00)	1.01 (1.00 – 1.03)	**1.08 (1.07 – 1.10)**	**0.97 (0.96 – 0.98)**	0.99 (0.98 – 1.01)	**1.07 (1.05 – 1.08)**
**Partnership status (ref: married/cohabiting)**									
Single	**0.98 (0.97 – 0.98)**	0.99 (0.98 – 1.00)	**0.97 (0.96 – 0.97)**	**1.02 (1.01 – 1.03)**	**1.02 (1.01 – 1.03)**	**0.98 (0.97 – 0.99)**	**0.95 (0.94 – 0.96)**	**0.96 (0.95 – 0.97)**	**0.96 (0.95 – 0.97)**
**Branch of industry (ref: services)**									
Manufacturing	**0.86 (0.85 – 0.87)**		**0.87 (0.86 – 0.88)**	**0.82 (0.80 – 0.84)**		**0.82 (0.80 – 0.84)**	**0.86 (0.84 – 0.87)**		**0.88 (0.87 – 0.89)**
Hospitality	0.99 (0.98 – 1.01)		1.02 (1.00 – 1.03)	1.01 (0.99 – 1.03)		1.02 (1.00 – 1.05)	0.99 (0.97 – 1.01)		1.01 (0.99 – 1.04)
Transport	**1.14 (1.11 – 1.16)**		**1.16 (1.13 – 1.18)**	**1.17 (1.10 – 1.24)**		**1.19 (1.12 – 1.26)**	**1.11 (1.08 – 1.13)**		**1.14 (1.12 – 1.17)**
Construction/installation	0.99 (0.97 – 1.01)		1.01 (1.00 – 1.03)	**1.12 (1.06 – 1.18)**		**1.12 (1.07 – 1.18)**	**0.95 (0.94 – 0.97)**		0.99 (0.98 – 1.01)
Care/education	**1.05 (1.04 – 1.06)**		**1.06 (1.05 – 1.07)**	**1.06 (1.05 – 1.08)**		**1.07 (1.06 – 1.09)**	**1.12 (1.10 – 1.14)**		**1.10 (1.08 – 1.12)**
Unknown	**0.38 (0.37 – 0.38)**		**0.38 (0.38 – 0.38)**	**0.40 (0.40 – 0.41)**		**0.40 (0.40 – 0.41)**	**0.36 (0.35 – 0.36)**		**0.36 (0.36 – 0.37)**
	**All in cohort70[e]**			**Women in cohort70[e]**			**Men in cohort70[e]**		
	**Crude TR (95% CI)**	**Model 1**	**Model 2**	**Crude TR (95% CI)**	**Model 1**	**Model 2**	**Crude TR (95% CI)**	**Model 1**	**Model 2**
**Mental SADP (ref: no mental SADP)[b]**									

Yes (any mental SADP)[c]	1.18 (0.97 – 1.44)	1.15 (0.94 – 1.39)	1.07 (0.88 – 1.30)	1.06 (0.83 – 1.36)	1.02 (0.80 – 1.31)	0.94 (0.74 – 1.20)	1.39 (1.00 – 1.93)	1.35 (0.97 – 1.87)	1.30 (0.95 – 1.79)
Number of days									
>0-30	1.30 (0.85 – 1.99)	1.27 (0.83 – 1.94)	1.13 (0.75 – 1.71)	1.16 (0.71 – 1.89)	1.11 (0.68 – 1.80)	0.99 (0.62 – 1.58)	1.78 (0.72 – 4.38)	1.75 (0.71 – 4.30)	1.60 (0.67 – 3.80)
>30-90	1.31 (0.89 – 1.93)	1.27 (0.86 – 1.86)	1.21 (0.84 – 1.76)	1.26 (0.78 – 2.06)	1.21 (0.74 – 1.96)	1.06 (0.66 – 1.71)	1.41 (0.75 – 2.68)	1.37 (0.72 – 2.58)	1.50 (0.81 – 2.77)
>90-180	1.35 (0.76 – 2.39)	1.32 (0.74 – 2.33)	1.26 (0.72 – 2.19)	2.32 (0.64 – 8.44)	2.25 (0.62 – 8.14)	2.28 (0.66 – 7.94)	1.05 (0.56 – 1.99)	1.03 (0.55 – 1.94)	0.91 (0.49 – 1.68)
>180	0.83 (0.47 – 1.47)	0.80 (0.45 – 1.42)	0.74 (0.43 – 1.29)	0.77 (0.36 – 1.61)	0.71 (0.34 – 1.50)	0.69 (0.34 – 1.43)	0.93 (0.38 – 2.28)	0.92 (0.37 – 2.25)	0.80 (0.34 – 1.91)
**Age (ref: 70-74)**									
≥75	**0.87 (0.86 – 0.88)**	**0.87 (0.86 – 0.88)**	**0.91 (0.90 – 0.92)**	**0.86 (0.84 – 0.88)**	**0.86 (0.84 – 0.88)**	**0.92 (0.90 – 0.94)**	**0.87 (0.86 – 0.89)**	**0.88 (0.86 – 0.89)**	**0.91 (0.89 – 0.92)**
**Sex (ref: men)**									
Women	**0.97 (0.96 – 0.98)**	**0.96 (0.95 – 0.97)**	0.99 (0.98 – 1.01)						
**Educational level (ref: university/college)**									
Elementary	**0.93 (0.92 – 0.95)**	**0.95 (0.94 – 0.97)**	**0.95 (0.94 – 0.97)**	**0.95 (0.93 – 0.98)**	0.98 (0.96 – 1.01)	0.99 (0.97 – 1.02)	**0.91 (0.90 – 0.93)**	**0.94 (0.92 – 0.96)**	**0.93 (0.91 – 0.95)**
High school	**0.96 (0.95 – 0.97)**	**0.97 (0.96 – 0.98)**	**0.98 (0.96 – 0.99)**	0.98 (0.96 – 1.00)	1.00 (0.97 – 1.02)	1.00 (0.98 – 1.02)	**0.94 (0.92 – 0.96)**	**0.95 (0.93 – 0.97)**	**0.96 (0.94 – 0.97)**
**Birth country (ref: Sweden)**									
Other	1.03 (1.00 – 1.05)	1.01 (0.99 – 1.03)	0.98 (0.96 – 1.00)	1.03 (1.00 – 1.07)	1.02 (0.98 – 1.06)	0.99 (0.96 – 1.03)	1.02 (1.00 – 1.05)	1.01 (0.98 – 1.04)	0.98 (0.95 – 1.01)
**Living area (ref: big city)**									
Medium-size city	**0.96 (0.94 – 0.97)**	**0.96 (0.95 – 0.98)**	**1.02 (1.01 – 1.03)**	**0.96 (0.94 – 0.98)**	**0.96 (0.94 – 0.98)**	1.03 (1.00 – 1.05)	**0.95 (0.94 – 0.97)**	**0.97 (0.95 – 0.98)**	1.01 (1.00 – 1.03)
Rural area	**0.92 (0.91 – 0.93)**	**0.94 (0.92 – 0.95)**	1.01 (1.00 – 1.03)	**0.92 (0.89 – 0.94)**	**0.93 (0.90 – 0.95)**	1.01 (0.99 – 1.04)	**0.92 (0.90 – 0.94)**	**0.94 (0.92 – 0.96)**	1.01 (0.99 – 1.03)
**Partnership status (ref: married/cohabiting)**									
Single	**0.98 (0.97 – 0.99)**	0.99 (0.98 – 1.00)	**0.98 (0.97 – 0.99)**	1.00 (0.98 – 1.02)	1.01 (0.99 – 1.02)	0.99 (0.97 – 1.00)	**0.97 (0.96 – 0.99)**	0.98 (0.96 – 1.00)	**0.98 (0.96 – 0.99)**
**Branch of industry (ref: services)**									
Manufacturing	**0.82 (0.80 – 0.83)**		**0.83 (0.82 – 0.85)**	**0.75 (0.73 – 0.78)**		**0.76 (0.74 – 0.79)**	**0.83 (0.82 – 0.85)**		**0.86 (0.84 – 0.88)**
Hospitality	1.02 (1.00 – 1.05)		1.03 (1.00 – 1.06)	1.02 (0.98 – 1.07)		1.02 (0.98 – 1.06)	1.03 (0.99 – 1.06)		**1.04 (1.01 – 1.08)**
Transport	**1.09 (1.06 – 1.13)**		**1.10 (1.06 – 1.13)**	**1.18 (1.08 – 1.30)**		**1.18 (1.07 – 1.29)**	**1.07 (1.03 – 1.11)**		**1.09 (1.05 – 1.12)**
Construction/installation	**0.95 (0.93 – 0.98)**		**0.96 (0.93 – 0.98)**	1.06 (0.98 – 1.14)		1.05 (0.97 – 1.13)	**0.93 (0.90 – 0.96)**		**0.94 (0.91 – 0.97)**
Care/education	**1.06 (1.05 – 1.08)**		**1.05 (1.04 – 1.07)**	**1.07 (1.04 – 1.09)**		**1.06 (1.03 – 1.09)**	**1.09 (1.06 – 1.12)**		**1.07 (1.04 – 1.10)**
Unknown	**0.54 (0.53 – 0.54)**		**0.54 (0.53 – 0.55)**	**0.57 (0.56 – 0.59)**		**0.57 (0.56 – 0.59)**	**0.51 (0.50 – 0.52)**		**0.51 (0.50 – 0.52)**

Note. [a]cohort65 = aged 65-69 in 2014, [b]Exposure variable, [c]Binary variable (yes/no), [d]Measured as a continuous variable from 65 to 69, [e]cohort70 = aged ≥70 in 2014. TR = Time Ratio. 95% CI = 95% Confidence Interval. Significant associations are in bold. TRs below 1 indicate shorter time until work exit, and TRs above 1 longer time until work exit. Model 1 was adjusted for age, sex, educational level, birth country, living area, and partnership status. Model 2 was adjusted for the same variables as Model 1 and branch of industry. The estimates for covariates were obtained from the model in which SADP was categorised by the number of days and were almost identical to those from the model where SADP was measured as yes/no. For mental SADP, the scale parameter was 0.58 for women and 0.57 for men in cohort65, and 0.63 for both women and men in cohort70.

Supplementary Table 3

Time Ratios for Somatic Sickness Absence and/or Disability Pension (SADP) and Time Until Work Exit, with Estimates for Covariates

	All in cohort65[a]			Women in cohort65[a]			Men in cohort65[a]		
	**Crude TR (95% CI)**	**Model 1**	**Model 2**	**Crude TR (95% CI)**	**Model 1**	**Model 2**	**Crude TR (95% CI)**	**Model 1**	**Model 2**
**Somatic SADP (ref: no somatic SADP)[b]**									
Yes (any somatic SADP)[c]	**0.90 (0.89 – 0.91)**	**0.93 (0.92 – 0.94)**	**0.94 (0.94 – 0.95)**	**0.92 (0.91 – 0.93)**	**0.94 (0.93 – 0.95)**	**0.95 (0.94 – 0.96)**	**0.89 (0.88 – 0.90)**	**0.92 (0.91 – 0.93)**	**0.94 (0.93 – 0.95)**
Number of days									
>0-30	**0.91 (0.89 – 0.92)**	**0.94 (0.93 – 0.96)**	**0.94 (0.92 – 0.95)**	**0.93 (0.91 – 0.95)**	**0.95 (0.93 – 0.98)**	**0.95 (0.93 – 0.96)**	**0.90 (0.88 – 0.92)**	**0.92 (0.90 – 0.95)**	**0.93 (0.91 – 0.95)**
>30-90	**0.92 (0.91 – 0.93)**	**0.95 (0.94 – 0.96)**	**0.94 (0.93 – 0.95)**	**0.94 (0.92 – 0.96)**	**0.96 (0.95 – 0.98)**	**0.95 (0.93 – 0.97)**	**0.91 (0.89 – 0.92)**	**0.93 (0.91 – 0.95)**	**0.93 (0.91 – 0.94)**
>90-180	**0.91 (0.89 – 0.92)**	**0.93 (0.91 – 0.95)**	**0.94 (0.92 – 0.95)**	**0.91 (0.89 – 0.94)**	**0.93 (0.90 – 0.95)**	**0.93 (0.91 – 0.95)**	**0.91 (0.88 – 0.93)**	**0.93 (0.91 – 0.96)**	**0.95 (0.92 – 0.97)**
>180-365	**0.90 (0.88 – 0.92)**	**0.92 (0.90 – 0.94)**	**0.93 (0.91 – 0.95)**	**0.93 (0.91 – 0.96)**	**0.94 (0.91 – 0.97)**	**0.94 (0.92 – 0.97)**	**0.88 (0.85 – 0.90)**	**0.89 (0.87 – 0.92)**	**0.92 (0.90 – 0.94)**
>365-731	**0.87 (0.86 – 0.89)**	**0.91 (0.89 – 0.93)**	**0.96 (0.94 – 0.98)**	**0.88 (0.86 – 0.91)**	**0.92 (0.89 – 0.94)**	**0.96 (0.94 – 0.99)**	**0.88 (0.85 – 0.91)**	**0.91 (0.88 – 0.94)**	**0.96 (0.93 – 0.99)**
>731	**0.84 (0.81 – 0.86)**	**0.91 (0.89 – 0.93)**	0.99 (0.97 – 1.02)	**0.84 (0.81 – 0.87)**	**0.92 (0.89 – 0.95)**	1.00 (0.97 – 1.04)	**0.84 (0.81 – 0.88)**	**0.90 (0.87 – 0.94)**	0.98 (0.95 – 1.01)
**Age[d]**	**1.06 (1.06 – 1.07)**	**1.06 (1.06 – 1.07)**	**1.07 (1.06 – 1.07)**	**1.07 (1.07 – 1.08)**	**1.07 (1.07 – 1.08)**	**1.08 (1.08 – 1.08)**	**1.06 (1.05 – 1.06)**	**1.06 (1.05 – 1.06)**	**1.06 (1.05 – 1.06)**
**Sex (ref: men)**									
Women	**0.93 (0.93 – 0.94)**	**0.94 (0.93 – 0.95)**	**0.98 (0.97 – 0.99)**						
**Educational level (ref: university/college)**									
Elementary	**0.90 (0.89 – 0.91)**	**0.89 (0.88 – 0.90)**	**0.90 (0.90 – 0.91)**	**0.87 (0.85 – 0.88)**	**0.86 (0.84 – 0.87)**	**0.92 (0.90 – 0.93)**	**0.90 (0.89 – 0.91)**	**0.91 (0.90 – 0.92)**	**0.89 (0.88 – 0.91)**
High school	**0.90 (0.89 – 0.91)**	**0.90 (0.90 – 0.91)**	**0.93 (0.92 – 0.93)**	**0.91 (0.90 – 0.92)**	**0.91 (0.90 – 0.92)**	**0.95 (0.94 – 0.96)**	**0.89 (0.88 – 0.90)**	**0.90 (0.89 – 0.91)**	**0.91 (0.90 – 0.92)**
**Birth country (ref: Sweden)**									
Other	1.01 (1.00 – 1.03)	**1.03 (1.01 – 1.04)**	1.01 (1.00 – 1.02)	**1.03 (1.01 – 1.05)**	**1.04 (1.02 – 1.06)**	**1.02 (1.01 – 1.04)**	1.01 (0.99 – 1.03)	1.01 (0.99 – 1.03)	1.00 (0.98 – 1.02)
**Living area (ref: big city)**									
Medium-size city	**0.96 (0.95 – 0.97)**	**0.98 (0.97 – 0.99)**	**1.03 (1.02 – 1.04)**	**0.97 (0.96 – 0.99)**	1.00 (0.98 – 1.01)	**1.05 (1.04 – 1.06)**	**0.95 (0.94 – 0.96)**	**0.96 (0.95 – 0.98)**	1.01 (1.00 – 1.02)
Rural area	**0.98 (0.97 – 0.99)**	1.00 (0.99 – 1.01)	**1.07 (1.06 – 1.08)**	0.98 (0.97 – 1.00)	1.01 (1.00 – 1.03)	**1.08 (1.07 – 1.10)**	**0.97 (0.96 – 0.98)**	1.00 (0.98 – 1.01)	**1.07 (1.06 – 1.08)**
**Partnership status (ref: married/cohabiting)**									
Single	**0.98 (0.97 – 0.98)**	0.99 (0.98 – 1.00)	**0.97 (0.96 – 0.97)**	**1.02 (1.01 – 1.03)**	**1.02 (1.01 – 1.03)**	**0.98 (0.97 – 0.99)**	**0.95 (0.94 – 0.96)**	**0.96 (0.95 – 0.97)**	**0.96 (0.95 – 0.97)**
**Branch of industry (ref: services)**									
Manufacturing	**0.86 (0.85 – 0.87)**		**0.87 (0.86 – 0.88)**	**0.82 (0.80 – 0.84)**		**0.82 (0.80 – 0.84)**	**0.86 (0.84 – 0.87)**		**0.88 (0.87 – 0.89)**
Hospitality	0.99 (0.98 – 1.01)		1.01 (1.00 – 1.03)	1.01 (0.99 – 1.03)		1.02 (1.00 – 1.05)	0.99 (0.97 – 1.01)		1.01 (0.99 – 1.04)
Transport	**1.14 (1.11 – 1.16)**		**1.16 (1.14 – 1.18)**	**1.17 (1.10 – 1.24)**		**1.18 (1.11 – 1.25)**	**1.11 (1.08 – 1.13)**		**1.15 (1.12 – 1.17)**
Construction/installation	0.99 (0.97 – 1.01)		1.02 (1.00 – 1.03)	**1.12 (1.06 – 1.18)**		**1.12 (1.06 – 1.17)**	**0.95 (0.94 – 0.97)**		1.00 (0.98 – 1.02)
Care/education	**1.05 (1.04 – 1.06)**		**1.07 (1.06 – 1.08)**	**1.06 (1.05 – 1.08)**		**1.08 (1.06 – 1.09)**	**1.12 (1.10 – 1.14)**		**1.10 (1.08 – 1.12)**
Unknown	**0.38 (0.37 – 0.38)**		**0.38 (0.38 – 0.38)**	**0.40 (0.40 – 0.41)**		**0.40 (0.40 – 0.41)**	**0.36 (0.35 – 0.36)**		**0.36 (0.36 – 0.37)**
	**All in cohort70[e]**			**Women in cohort70[e]**			**Men in cohort70[e]**		
	**Crude TR (95% CI)**	**Model 1**	**Model 2**	**Crude TR (95% CI)**	**Model 1**	**Model 2**	**Crude TR (95% CI)**	**Model 1**	**Model 2**
**Somatic SADP (ref: no somatic SADP)[b]**									

Yes (any somatic SADP)[c]	**1.12 (1.08 – 1.16)**	**1.10 (1.06 – 1.14)**	**1.05 (1.01 – 1.08)**	**1.14 (1.07 – 1.20)**	**1.11 (1.05 – 1.17)**	1.05 (0.99 – 1.11)	**1.11 (1.06 – 1.16)**	**1.09 (1.04 – 1.14)**	1.04 (1.00 – 1.09)
Number of days									
>0-30	**1.16 (1.08 – 1.25)**	**1.13 (1.05 – 1.22)**	1.07 (1.00 – 1.15)	**1.21 (1.09 – 1.36)**	**1.18 (1.06 – 1.32)**	**1.13 (1.01 – 1.26)**	**1.12 (1.01 – 1.23)**	1.09 (0.99 – 1.20)	1.03 (0.94 – 1.13)
>30-90	**1.13 (1.07 – 1.19)**	**1.11 (1.05 – 1.17)**	1.04 (0.99 – 1.10)	**1.11 (1.03 – 1.21)**	1.09 (1.00 – 1.19)	1.03 (0.95 – 1.11)	**1.14 (1.06 – 1.23)**	**1.12 (1.04 – 1.20)**	1.05 (0.98 – 1.13)
>90-180	**1.13 (1.05 – 1.21)**	**1.11 (1.03 – 1.20)**	1.08 (1.00 – 1.15)	**1.17 (1.04 – 1.33)**	**1.15 (1.02 – 1.30)**	1.08 (0.96 – 1.21)	**1.10 (1.01 – 1.21)**	1.09 (1.00 – 1.19)	1.07 (0.98 – 1.17)
>180	0.99 (0.89 – 1.09)	0.97 (0.88 – 1.07)	0.95 (0.86 – 1.05)	0.91 (0.75 – 1.11)	0.89 (0.73 – 1.08)	0.86 (0.72 – 1.04)	1.01 (0.90 – 1.13)	1.00 (0.89 – 1.12)	0.99 (0.88 – 1.10)
**Age (ref: 70-74)**									
≥75	**0.87 (0.86 – 0.88)**	**0.87 (0.86 – 0.89)**	**0.91 (0.90 – 0.92)**	**0.86 (0.84 – 0.88)**	**0.86 (0.84 – 0.88)**	**0.92 (0.90 – 0.94)**	**0.87 (0.86 – 0.89)**	**0.88 (0.86 – 0.89)**	**0.91 (0.89 – 0.92)**
**Sex (ref: men)**									
Women	**0.97 (0.96 – 0.98)**	**0.96 (0.95 – 0.97)**	0.99 (0.98 – 1.00)						
**Educational level (ref: university/college)**									
Elementary	**0.93 (0.92 – 0.95)**	**0.95 (0.94 – 0.97)**	**0.95 (0.94 – 0.97)**	**0.95 (0.93 – 0.98)**	0.98 (0.96 – 1.01)	0.99 (0.97 – 1.02)	**0.91 (0.90 – 0.93)**	**0.94 (0.92 – 0.96)**	**0.93 (0.91 – 0.95)**
High school	**0.96 (0.95 – 0.97)**	**0.97 (0.96 – 0.98)**	**0.98 (0.96 – 0.99)**	0.98 (0.96 – 1.00)	1.00 (0.97 – 1.02)	1.00 (0.98 – 1.02)	**0.94 (0.92 – 0.96)**	**0.95 (0.93 – 0.97)**	**0.96 (0.94 – 0.97)**
**Birth country (ref: Sweden)**									
Other	1.03 (1.00 – 1.05)	1.01 (0.99 – 1.03)	0.98 (0.96 – 1.00)	1.03 (1.00 – 1.07)	1.02 (0.98 – 1.06)	0.99 (0.96 – 1.02)	1.02 (1.00 – 1.05)	1.01 (0.98 – 1.03)	0.98 (0.95 – 1.00)
**Living area (ref: big city)**									
Medium-size city	**0.96 (0.94 – 0.97)**	**0.97 (0.95 – 0.98)**	**1.02 (1.01 – 1.03)**	**0.96 (0.94 – 0.98)**	**0.96 (0.94 – 0.98)**	1.03 (1.00 – 1.05)	**0.95 (0.94 – 0.97)**	**0.97 (0.95 – 0.98)**	1.02 (1.00 – 1.03)
Rural area	**0.92 (0.91 – 0.93)**	**0.94 (0.92 – 0.95)**	1.01 (1.00 – 1.03)	**0.92 (0.89 – 0.94)**	**0.93 (0.90 – 0.95)**	1.01 (0.99 – 1.04)	**0.92 (0.90 – 0.94)**	**0.94 (0.92 – 0.96)**	1.01 (1.00 – 1.03)
**Partnership status (ref: married/cohabiting)**									
Single	**0.98 (0.97 – 0.99)**	0.99 (0.98 – 1.00)	**0.98 (0.97 – 0.99)**	1.00 (0.98 – 1.02)	1.00 (0.99 – 1.02)	0.98 (0.97 – 1.00)	**0.97 (0.96 – 0.99)**	0.98 (0.96 – 1.00)	**0.98 (0.96 – 0.99)**
**Branch of industry (ref: services)**									
Manufacturing	**0.82 (0.80 – 0.83)**		**0.83 (0.82 – 0.85)**	**0.75 (0.73 – 0.78)**		**0.76 (0.74 – 0.79)**	**0.83 (0.82 – 0.85)**		**0.86 (0.84 – 0.88)**
Hospitality	1.02 (1.00 – 1.05)		1.03 (1.00 – 1.06)	1.02 (0.98 – 1.07)		1.02 (0.98 – 1.06)	1.03 (0.99 – 1.06)		**1.04 (1.01 – 1.08)**
Transport	**1.09 (1.06 – 1.13)**		**1.09 (1.06 – 1.13)**	**1.18 (1.08 – 1.30)**		**1.17 (1.07 – 1.29)**	**1.07 (1.03 – 1.11)**		**1.09 (1.05 – 1.12)**
Construction/installation	**0.95 (0.93 – 0.98)**		**0.96 (0.93 – 0.98)**	1.06 (0.98 – 1.14)		1.05 (0.97 – 1.13)	**0.93 (0.90 – 0.96)**		**0.94 (0.91 – 0.97)**
Care/education	**1.06 (1.05 – 1.08)**		**1.05 (1.03 – 1.07)**	**1.07 (1.04 – 1.09)**		**1.06 (1.03 – 1.08)**	**1.09 (1.06 – 1.12)**		**1.07 (1.04 – 1.10)**
Unknown	**0.54 (0.53 – 0.54)**		**0.54 (0.53 – 0.55)**	**0.57 (0.56 – 0.59)**		**0.57 (0.56 – 0.59)**	**0.51 (0.50 – 0.52)**		**0.51 (0.50 – 0.52)**

Note. [a]cohort65 = aged 65-69 in 2014, [b]Exposure variable, [c]Binary variable (yes/no), [d]Measured as a continuous variable from 65 to 69, [e]cohort70 = aged ≥70 in 2014. TR = Time Ratio. 95% CI = 95% Confidence Interval. Significant associations are in bold. TRs below 1 indicate shorter time until work exit, and TRs above 1 longer time until work exit. Model 1 was adjusted for age, sex, educational level, birth country, living area, and partnership status. Model 2 was adjusted for the same variables as Model 1 and branch of industry. The estimates for covariates were obtained from the model in which SADP was categorised by the number of days and were almost identical to those from the model where SADP was measured as yes/no. For somatic SADP, the scale parameter was 0.58 for women and 0.57 for men in cohort65, and 0.64 for women and 0.63 for men in cohort70.
